# Monoclonal gammopathy of undetermined significance: evaluation, risk assessment, management, and beyond

**DOI:** 10.12703/r/11-34

**Published:** 2022-11-29

**Authors:** Jithma P Abeykoon, Reema K Tawfiq, Shaji Kumar, Stephen M Ansell

**Affiliations:** 1 Division of Hematology, Mayo Clinic, 200 First Street SW, Rochester, MN 55905, USA; 2 Department of Internal Medicine, Mayo Clinic, Rochester, MN, USA

**Keywords:** Cancer, screening, pre-malignant, multiple myeloma, plasma cell dyscrasia, malignancy

## Abstract

Monoclonal gammopathy of undetermined significance (MGUS) is a premalignant state for a spectrum of lymphoplasmacytic malignancies. The risk of progression of MGUS to a symptomatic therapy requiring plasma cell dyscrasia is about 1% per year. Studies carried out over the previous 10 years have improved risk stratification of MGUS based on serologic and genomic evaluations, which has led to better management of patients. In this review, we address the epidemiology, diagnosis, and pathogenesis of MGUS and discuss risk-adapted best practice approaches to monitor patients.

## Introduction

Monoclonal gammopathy (MG) encompasses several conditions defined by the increased proliferation of a clone of plasma cells that produce an abundance of a monoclonal immunoglobulin: monoclonal (M) protein. Within this category fall both benign hematologic conditions such as monoclonal gammopathy of undetermined significance (MGUS) and lymphoplasmacytic malignancies (LPMs), including multiple myeloma (MM), Waldenström macroglobulinemia (WM), and amyloid light-chain amyloidosis (AL)^1^. Though fundamentally similar, each MG has its unique symptoms, diagnostic criteria, treatments, and disease trajectories. The focus of this review is to showcase the recent advances in the diagnosis, risk stratification, and management of MGUS.

MGUS was first described in 1960 by Jan Waldenström as “essential hyperglobulinemia” or “benign monoclonal gammopathy.” He firmly believed that benign MG was unrelated to MM^2^. However, in 1978, Robert Kyle coined the current term “monoclonal gammopathy of undetermined significance” after his observational retrospective study of 241 patients showed that some patients with MGUS progressed to MM, WM, or AL^3^. Since that time, population studies from Olmsted County (Minnesota) and Ghana, as well as the National Health and Nutrition Examination Survey (NHANES) in the United States and the Prostate, Lung, Colorectal, And Ovarian (PLCO) cancer screening trial, have significantly advanced our knowledge of MGUS and the disorders it precedes^4–8^.

### Definition

MGUS is a clinically asymptomatic, premalignant, clonal plasma cell disorder and is an obligatory precursor for several LPMs, including MM, WM, and AL^9^. It is defined by the presence of serum or urine M protein, <10% clonal plasma cells in the bone marrow, and the absence of a diagnosis of MM or related LPMs^1^. Three distinct subtypes of MGUS are classified based on the M protein isotype: immunoglobulin M (IgM) MGUS, non-IgM (IgG, IgA, or IgD) MGUS, and light-chain MGUS (LC-MGUS). The risk of progression into an LPM is different for each of these subtypes of MGUS^9,10^.

### Epidemiology

The prevalence of MGUS increases with age and is observed in nearly 3% of the population ≥50 years old and 5% of those ≥70 years^4,11^. For patients aged <40 years, MGUS is a relatively rare event with a prevalence of <0.3%, and this patient population represents only 2% of all patients with MGUS^12^. The incidence and prevalence of MGUS are also higher in men than women and are two- to three-fold higher in Blacks than Whites. The incidence of MGUS in men is 120 per 100,000 at age 50 and increases to 530 per 100,000 by the age of 90^13^. The corresponding rates for women are 60 per 100,000 population at age 50 and 370 per 100,000 at age 90^13^. The prevalences of MGUS among a well-defined, predominantly White population in persons ≥50, ≥70, and ≥85 years of age are estimated to be 3.2%, 5.3%, and 7.5%, respectively^4^. The age-adjusted prevalences of MGUS using population-based racial surveys are 3.7%, 2.3%, 1.8%, and 2.1% in Blacks, Whites, Hispanics, and Japanese, respectively^5,7,14,15^. Table 1 outlines the prevalence of MGUS in different countries based on population studies. Furthermore, there is a higher risk of an earlier age of onset in Blacks than Whites^6^. In a recent population-based analysis, the prevalence of MGUS detected using matrix-assisted laser desorption ionization time-of-flight mass spectrometry (MALDI-TOF MS) was 17% in Blacks who are >50 years old^16^. In that study, high-risk individuals were categorized as Black patients and those with first-degree relatives with a diagnosed hematologic malignancy, and the prevalences of MGUS were 4.9% and 13% in patients between the ages of 40 and 49 and between 70 and 79, respectively^16^.

### Pathogenesis and genomic landscape

Plasma cells are terminally differentiated B lymphocytes derived from post-germinal center B cells^17^. Owing to defined genomic aberrations, monoclonal plasma cell populations emerge from a polyclonal background, and with fluorescence *in situ* hybridization (FISH), chromosomal abnormalities have been identified in the development of MGUS^18–20^. About 50% of patients with MGUS demonstrate aneuploidy, particularly hyperploidy. This is compared with chromosome aneuploidy seen in about 75% of patients with MM or LPM^21^. Translocations involving the immunoglobulin heavy chain (IGH) locus on chromosome 14q32 and one of five partner chromosomes — 11q13 (*cyclin D1* gene, the most common), 4p16.3 (*FGFR-3* and *MMSET*), 6p21 (*cyclin D3* gene), 16q23 (*c-maf*), and 20q11 (*mafB*) — have been identified in close to half of the patients with MGUS (Table 2)^10,22^. In addition to IGH translocations, known myeloma-specific chromosome abnormalities have been detected in MGUS, such as *RB1* (13q14) deletion, 1q-gain, and hyperdiploidy; however, the frequency of these abnormalities is lower in MGUS than in MM^23,24^. Consistent with the primary lesion hypothesis, one study found IGH translocations comprising (4;14), t(11;14), t(14;16), and t(14;20) to be around 27%, which is similar to that seen in MM. In another study, t(11;14), t(4;14), t(14;16), t(14;20), and t(6;14) were seen between 0.5% and 16% of patients with MGUS^18^. Moreover, comparable to myeloma, mutations in genes such as *RAS*, *NRAS*, *DIS3*, *HIST1H1E*, *EGR1*, and *LTB* were also found in MGUS^25,26^. Importantly, mutations or deletions of *TP53* and *MYC* translocations were not detected in patients with MGUS as one may see in MM or LPM, suggesting that these events could happen later in the disease course and may lead to MGUS progression to more advanced stages^25^. The genetic alterations in IgM MGUS, unlike those in non-IgM MGUS, seem to be different, where *MYD88*^L265P^ and *CXCR4* were the main mutations seen in 60% and 9%, respectively^27^. These cytogenetic changes could be the primary events in initiating the process of plasma cell immortalization. Therefore, the fate of MGUS, as in its persistence as an indolent malignancy or its progression to aggressive cancer, is dictated by the different cytogenetic changes acquired by the plasma cell clone over time^10,28,29^.

**Table 1.** Prevalence of MGUS in population-based studies.

Location	Length of study	Number of persons studied	Number of MGUS cases	Overall prevalence (95% CI)	Median age (range)	Median age at diagnosis (range)	Reference
Olmsted County, Minnesota, USA	7 years	21,463 (9,469 ♂, 11,994 ♀)	694	3.2 (3.0–3.5)	70	NA	Kyle *et al*. (2006)^4^
National Health and Nutrition Examination Survey (NHANES), USA	6 years	12, 482 (6,069 ♂, 6,413 ♀)	365	2.4 (1.2–2.5)	71.8	NA	Landgren *et al*. (2014)^7^
Thailand (2 communities in Bangkok, 1 suburban community, and 2 in rural communities)	8 months	3,260 (1,104 ♂, 2,156 ♀)	75	2.3 (1.8–2.8)	57 (50–93)	58 (50–81)	Watanaboonyongcharoen *et al*. (2012)^30^
Beijing, China	5 years	154,597 (82,705 ♂, 71,892 ♀)	843	1.11 (1.0–1.2)	58 (25–96)	NA	Han *et al*. (2020)^31^
Hong Kong, China	1 year	1,000 (500 ♂, 500 ♀)	8	0.8 (0.3–1.4)	57 (50–65)	NA	Wu *et al*. (2013)^32^
Nagasaki City, Japan	15 years	52,802 (20,583 ♂, 32,219 ♀)	1,088	2.1 (1.9–2.2)	NA	68.5 (45–100)	Iwanaga *et al*. (2007)^15^
Seongnam-si, Seoul, Korea	1 year	680 (287 ♂, 393 ♀)	21	3.3 (1.9–4.6)	74.5 (66–92)	NA	Park *et al*. (2011)^33^
Ghana	2 years	917 (all men)	54	5.9 (4.4–7.4)	60 (50–74)	NA	Landgren *et al*. (2007)^5^
Heinz Nixdorf Recall Study, Germany	3 years	MGUS: 4,702 (2,363 ♂, 2,339 ♀) LC-MGUS: 4,695 (2,361 ♂, 2,334 ♀)	MGUS: 165 LC-MGUS: 34	MGUS: 3.5 (3.0–4.1) LC-MGUS: 0.7 (0.5–1.0)	MGUS: 63 (47–75) LC-MGUS: 67 (47–74)	NA	Eisele *et al*. (2012)^34^
Agriculture cohort from Calvados area in Normandy, France	3 years	775 (445 ♂, 320 ♀)	26	7.2 (NA)	♂: 44 (17–76) ♀: (46 (19–96)	NA	Lecluse *et al*. (2016)^35^

♂, male; ♀, female; CI, confidence interval; LC-MGUS, light-chain monoclonal gammopathy of undetermined significance; MGUS, monoclonal gammopathy of undetermined significance; NA, not available.

**Table 2.** Chromosomal aberrations in monoclonal gammopathy of undetermined significance compared to multiple myeloma.

Chromosomal aberrations	MGUS, %	MM, %
Normal cytogenetics	19	3
t(11;14) (q13;q32)	16	15–20
t(4;14) (p16;q32)	2	15
t(14;16) (q32;q23)	5	5
t(6;14) (p21;q32)	-	4
t(14;20) (q32;q11)	-	<1
Aneuploidy	50	75
Hyperdiploidy	54	59.5
Hypodiploidy	11.5	25

MGUS, monoclonal gammopathy of undetermined significance; MM, multiple myeloma.

The genomic landscape of myeloma precursor conditions such as MGUS and smoldering myeloma has expanded with the use of whole genome sequencing. A study by Oben *et al*. identified myeloma-defining genomic events, which include chromothripsis, templated insertions, mutations in driver genes, aneuploidy, and canonical apolipoprotein B mRNA-editing catalytic polypeptide (APOBEC) mutational activity to be associated with MGUS progression^36^. In that study, it was found that patients with MGUS without progression had a lower burden of myeloma-defining genomic events when compared with patients who had a high number of myeloma-defining genomic events in whom MGUS progression was imminent^36,37^.

Myeloma-defining genomic events include structural variants, alterations in driver genes, clonal IGH translocations, genomic copy number changes, hyperdiploidy, and MYC translocation^36–38^. The recent advances in molecular and genomic techniques, including whole exome sequencing (WES), single-nucleotide polymorphism array, and global gene expression profiling, have uncovered these myeloma-defining genomic events and have enhanced our understanding of the disease biology of MGUS, smoldering myeloma, and MM. Of these genomic aberrations, hyperdiploidy and canonical IGH translocations are seen more commonly in MGUS, but other genomic alterations are rare. Another study looked at the tumor mutational burden (TMB), single-base substitution (SBS), and activation-induced cytidine deaminase (AID)-induced somatic mutagenesis in MGUS and MM. In that study, the TMB and SBS were high in MM compared with MGUS, and the presence of the APOBEC signature was a predictor of poor overall survival in patients with MM. Additionally, patients with MM who had poor outcomes had high TMB compared with patients with better outcomes^39^. Detecting these genomic aberrations early in the clonal evolution of plasma cells has enabled us to better understand the progression of MGUS to MM^36,40^. The advent of genomic techniques may pave the path to better risk-stratify patients based on genomic alterations and predict disease progression before the appearance of clinical and laboratory indicators. However, these sophisticated genomic analysis techniques are not universally available, and most of the findings based on genomic alterations have yet to be prospectively validated. Hence, the practice patterns should still be based on clinical and laboratory parameters along with chromosomal alterations, which have already been established and incorporated in MM risk stratification models^36–41^.

## Diagnostic criteria and risk stratification

Based on the current diagnostic criteria from the 2014 International Myeloma Working Group (IMWG), MGUS is diagnosed when all three of the criteria are met: serum M protein less than 3 g/dL or the presence of abnormal free light chain (FLC) ratio, bone marrow plasma cells less than 10%, and the absence of end-organ damage attributed to plasma cells^1^. IgM MGUS is defined by serum IgM M protein, and non-IgM MGUS is characterized by serum IgG, IgA, and (rarely) IgD or IgE M proteins^1^. LC-MGUS is defined by the presence of an abnormal FLC ratio (<0.26 or >1.65; involved-to-uninvolved FLC ratio of less than 100 with a lack of IGH expression on immunofixation), increased level of involved FLC more than the upper limit of normal (ULN), urinary monoclonal protein of less than 500 mg/24 hours, and the presence of the aforementioned three criteria^1,42^.

The absence of end-organ damage translates to the lack of hypercalcemia, renal insufficiency, anemia, and bone lesions (referred to as CRAB features) attributable to an underlying plasma cell disorder. Hypercalcemia is defined by serum calcium greater than 1 mg/dL (>0.25 mmol/L) of ULN or higher than 11 mg/dL (>2.75 mmol/L). Renal insufficiency is defined as a creatinine clearance of less than 40 mL/min or a serum creatinine greater than 2 mg/dL. Anemia is defined by a hemoglobin value greater than 2 g/dL below the lower limit of normal or a value less than 10 g/dL. Bone lesions are osteolytic lesions that can be detected on skeletal radiography, computed tomography (CT), or positron emission tomography-CT^1^. Following the diagnosis of MGUS, risk stratification of patients should be done based on the presence or absence of risk factors that increase the rate of progression of MGUS to LPM.

## Risk and assessment of progression

**Figure 1.**
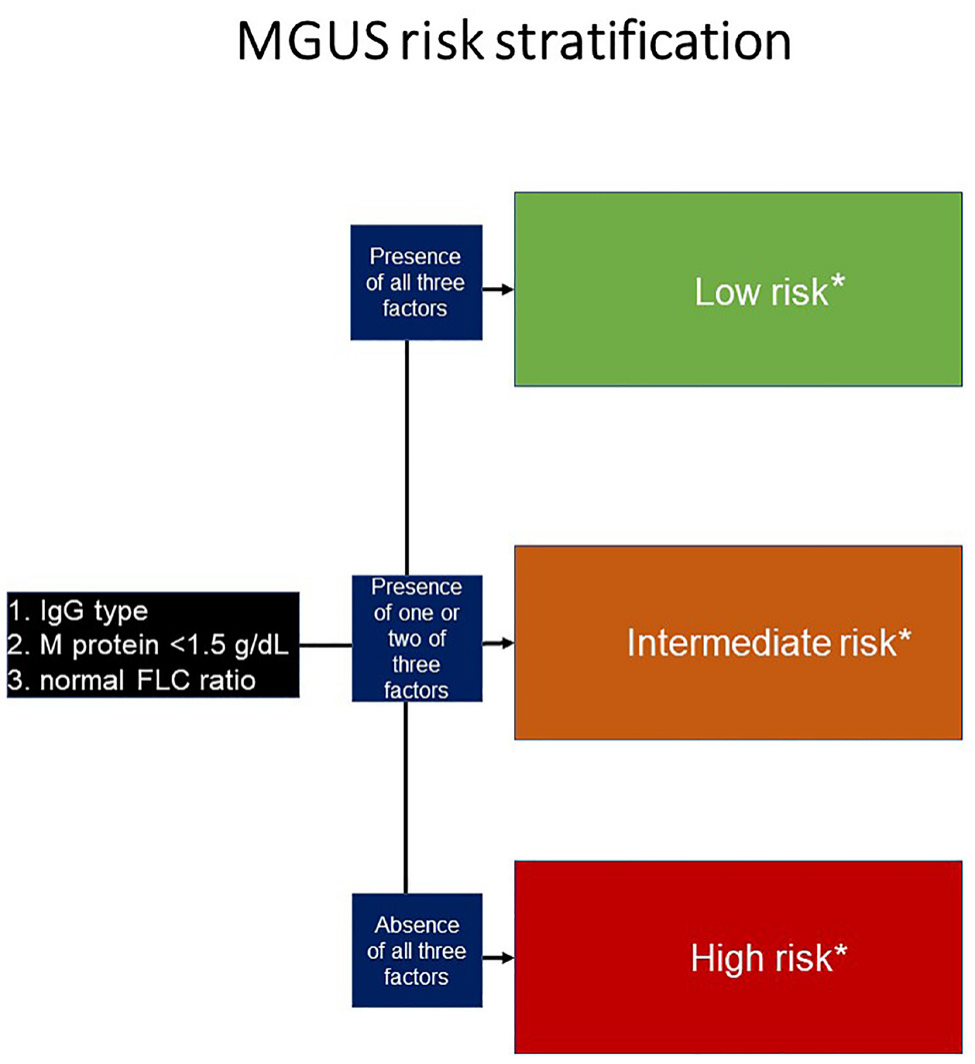
Mayo MGUS risk stratification. *Low risk: 5% risk of progression in 20 years; intermediate risk: 20% risk of progression in 20 years; high risk: 60% risk of progression in 20 years. FLC, free light chain ratio; IgG, immunoglobulin G; MGUS, monoclonal gammopathy of undetermined significance.

The rate of progression of MGUS to LPM is 0.5–1% per year, but the exact risk depends on the concentration and type of the M protein, serum FLC ratio, bone marrow plasmacytosis, proportion of phenotypically clonal plasma cells, and presence of immunoparesis^1^. The three major risk factors for the progression of MGUS are an abnormal serum FLC ratio (i.e., the ratio of free immunoglobulin κ to λ light chains in the serum), non-IgG MGUS, and a high serum M protein level (≥1.5 g/dL) (Figure 1)^11,43^. Based on the Mayo Clinic’s stratification model, the presence of all three factors constitutes high-risk MGUS. High-intermediate-risk MGUS is present if any two of the factors are present, low-intermediate-risk MGUS is present if any one of the three factors is present, and the absence of all three factors is classified as low-risk MGUS. The risk of progression to LPM at 20 years when one, two, and three risk factors are present is 5%, 21%, and 58%, respectively^11,43,44^.

IgM MGUS has a higher risk of progression than non-IgM and is typically associated with progression to lymphoplasmacytic lymphoma/WM^1,11^. The risk of progression among patients with IgM MGUS is 2% per year in the first 10 years after diagnosis and 1% per year thereafter^11^. In contrast, non-IgM MGUS is associated with a risk of progression to MM at a rate of 0.5–1% per year^1,11^. Though rare, both IgM MGUS and non-IgM MGUS can progress to AL, and the risk is about 1% over 35 years of follow-up^11^. LC-MGUS can progress to light-chain MM and AL with a risk of 0.3% per year^1^.

In addition to the aforementioned risk factors, a study by Kyle *et al*. found that the risk of progression was higher when there are >5% clonal plasma cells in the bone marrow and low concentrations of two uninvolved immunoglobulins^11^. However, this heightened risk was not observed with a lower concentration of only one uninvolved immunoglobulin level^11^. The age, sex, presence of hepatosplenomegaly, hemoglobin values, serum creatinine, serum albumin, and quantitative measurements of a monoclonal urinary light chain were not predictors of MGUS progression^11^.

Conversely, the Spanish study group recognized multiparametric flow cytometry as a valuable tool to identify aberrant plasma cell populations and predict the risk of MGUS progression to MM^45^. The antigens that are most frequently used to identify aberrant plasma cells include CD19, CD45, and CD56 in combination with CD38/CD138^46,47^. The validated immunophenotypic approach to identify aberrant phenotypes in plasma cells is the absence of CD19 or CD45, the decreased expression of CD38, and the overexpression of CD56^48,49^. According to the Spanish study group, the two risk factors for the progression of MGUS are the percentage of aberrant plasma cells to bone marrow plasma cells of above 95% and DNA aneuploidy. When these independent variables are used, the rates of progression-free survival at 5 years for MGUS patients with zero, one, and two risk factors are 2%, 10%, and 46%, respectively^45^.

Another study classified patients with MGUS into three risk categories based on the cytogenetic risk factors: high risk (t(4;14) and chromosome 17p deletion), intermediate risk (trisomies without translocations), and standard risk (t(11;14), all translocations other than t(4;14) and chromosome 13 abnormalities). In that study, the median time-to-progression of MGUS was 4.7 years for patients with high-risk cytogenetics and was not reached for other risk groups in a 4.2-year follow-up^18^.

Earlier onset of MGUS does not imply a more aggressive or indolent disease. Pang *et al.* showed that young patients with MGUS had an average progression rate of 1.4% per year, similar to older patients^12^. Furthermore, autoimmune diseases have been associated with an increased risk of MGUS development with a relative risk of 1.42, and the strongest association was seen with pernicious anemia with a relative risk of 1.67^50^. It has been hypothesized that the constant stimulation of the immune system in immune-related conditions can cause B-cell dysfunction and clonal plasma cell disorders^51^. Interestingly, although the prevalence of MGUS is increased with autoimmune conditions, patients with immune-related disorders may have a relatively lower risk of progression. In one study, the M protein level was found to be higher in patients without autoimmune conditions as compared with patients with autoimmune conditions^12^. However, in that study, the size of the M protein was the strongest risk factor, and the presence of an autoimmune condition by itself was not an independent risk factor for MGUS progression^12^.

In addition to these risk factors, one should pay close attention to clinical and laboratory “red flags” when following patients with MGUS (Table 3). In the presence of these “red flags” in the correct clinical setting without an alternative explanation, one should promptly evaluate patients for progression to an LPM^9^.

**Table 3.** Clinical and laboratory “red flags” that clinicians should be aware of while following patients with MGUS.

Clinical “red flags”	Laboratory “red flags”
Constitutional signs of malignancy, including excessive fatigue, drenching night sweats, fever, and unintentional weight loss	Anemia
Excessive bone pain or pathologic fractures	Elevated creatinine and renal impairment
Neuropathy	Hypercalcemia
Organomegaly and lymphadenopathy	Progression of serum M protein ≥50% or ≥3 g/dL with an absolute increase of ≥0.5 g/dL
Mucocutaneous bleeding	Progression of involved serum FLC ratio by ≥50% or the ratio of involved to uninvolved FLC ratio ≥100 when absolute FLC ratio at least 100 mg/L
	Urine protein electrophoresis showing urine M protein ≥500 mg in 24 hours

FLC, free light chain; M monoclonal; MGUS, monoclonal gammopathy of undetermined significance.

## Screening and indications for testing

Currently, owing to the lack of evidence supporting the clinical benefit of early detection, screening of asymptomatic MGUS in the general population is not recommended^11^. Therefore, MGUS is generally found incidentally while evaluating a patient with signs and symptoms suggestive of a possible LPM or any of the associated conditions. Clinicians also look for MG by testing for M protein in patients with a nonmalignant disease known to cause or be associated with MG^52,53^.

A recent study at the Mayo Clinic found that monoclonal protein testing is commonly performed for signs and symptoms not typically associated with LPM^54^. The top five indications for testing were neuropathy (19.8%), renal disease (13.7%), anemia (12.8%), bone disorders or connective tissue pain (12.8%), and cutaneous diseases (5.8%). The subsequent diagnoses of the common indications were neuropathy-no other source (NOS), chronic kidney disease-NOS, iron deficiency, and osteoporosis/osteopenia, respectively. In that study, neuropathy was associated with IgM MGUS in close to 19% of patients^54^. Initial screening of MGUS was predominantly done by general internal medicine (31.3%), neurology (10.3%), and cardiology (9.7%)^54^.

The Iceland Screens, Treats, or Prevents Multiple Myeloma (iStopMM) study is the first population-based, prospective screening study and randomized controlled trial based in Iceland to evaluate the potential harm and benefit of MGUS screening^55^. The recent results of the iStopMM study involving 75,422 total participants with a 5% prevalence of MGUS found that active screening of patients with MGUS could identify a higher number of patients with progression than patients who are followed by current established guidelines^56^. However, the study is still evolving, and results pertaining to the survival of these patients are pending. While these data are maturing, the study’s authors advised against preemptive MGUS screening in otherwise-healthy individuals^56^. In the meantime, experts believe that it may be beneficial to screen for MG in high-risk patients who have two or more first-degree relatives with MM, AL, or WM^9^.

At the time of initial evaluation of a patient with suspected MGUS, complete blood count, serum M protein, serum FLC, and IGH evaluation should be performed. Based on the 2018 publication by Go *et al.*^9^, in the absence of concerning clinical or laboratory features along with the presence of low-risk MGUS (IgG MGUS, serum M protein <1.5 mg/dL, and normal FLC ratio, if LC-MGUS FLC ratio <8), one could omit a bone marrow biopsy and skeletal survey as only 2% of these low-risk patients progress during their lifetime^44^.

## Management of patients after MGUS diagnosis

Currently, there is no role in initiating any disease-specific treatments with chemoimmunotherapy or targeted therapy for patients with MGUS. However, clinical follow-up in conjunction with focused laboratory evaluations based on the risk of progression as well as establishing patient expectations and proper education is paramount in managing patients with MGUS. The monitoring parameters for patients with MGUS should be based on the risk factors and “red flags” for the progression of MGUS. All patients should undergo repeat laboratory evaluations within 3–6 months from the time of initial MGUS diagnosis. These laboratory evaluations include complete blood count, serum protein electrophoresis, serum FLC assessment, and assessment of calcium and serum creatinine^9^.

The data for monitoring patients with MGUS are derived from a few retrospective and population-based studies. A study by Go *et al.*, including 17,963 patients with a follow-up of 46,276 person-years, identified that the majority of progression (2%) occurs within the first 2 years and gradually declines thereafter with a rate of 0.8% at 5 years^51^. The seminal study by Kyle *et al*. identified the risk of progression as 2% per year for the first 10 years, decreasing to 1% per year thereafter^11^. For patients with an abnormal FLC ratio and ≥1.5 g/dL serum M protein, 3.6% of patients per 100 person-years had MGUS progression, as compared with 1.1 per 100 person-years of patients in whom neither of these risk factors was seen^11^.

### Monitoring after MGUS diagnosis

Many of the governing bodies have put forward guidelines to monitor patients with MGUS. The UK Myeloma Forum/Nordic Myeloma Study Group has recommended monitoring patients with low-risk MGUS every 3–4 months for the first year and 6–12 months thereafter if no disease progression is detected. In the absence of low-risk MGUS (in other words, in higher-risk patients), patients should be followed every 3–4 months^57^. The International Expert Consensus has recommended monitoring patients every 4–6 months for the first 2 years and every 6–24 months thereafter for all patients with MGUS^58^. The European Myeloma Network recommends re-evaluating patients at 6 months from diagnosis and yearly thereafter. For low-risk patients, no follow-up is recommended if the disease is stable at 6 months from diagnosis^24^. Recommendations from the IMWG suggest following low-risk patients at 6 months from the diagnosis and then every 2–3 years if the disease is stable. For patients with higher-risk disease, follow-up should be done annually after an initial 6-month follow-up from diagnosis^59^.

**Figure 2.**
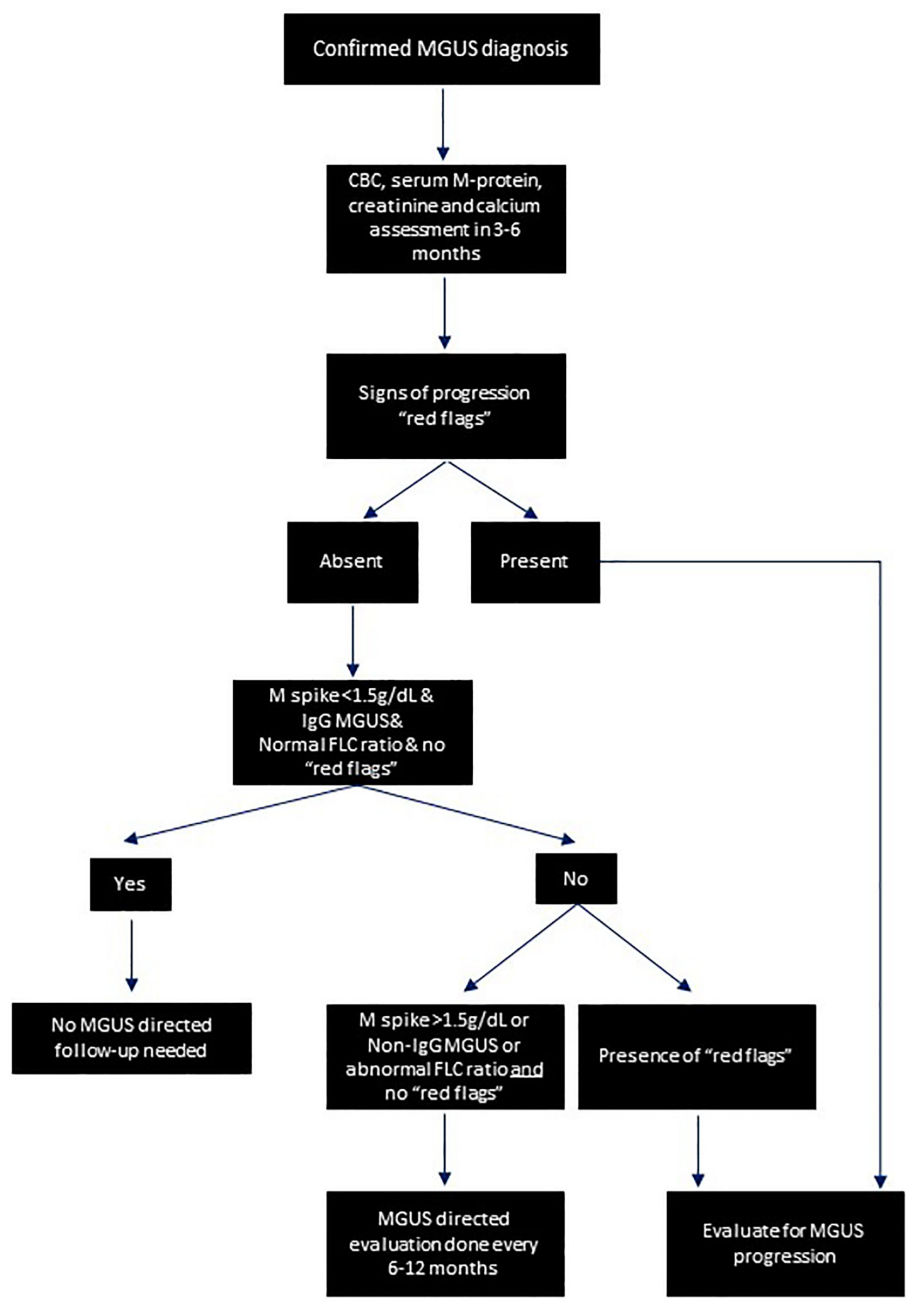
Suggested algorithm for monitoring patients with monoclonal gammopathy of undetermined significance. “Red flags”: constitutional signs of malignancy, including excessive fatigue, drenching night sweats, fever, unintentional weight loss, excessive bone pain or pathologic fractures, neuropathy, organomegaly and lymphadenopathy, mucocutaneous bleeding, unexplained anemia, elevated creatinine and renal impairment, unexplained hypercalcemia, progression of serum M protein ≥50% or ≥3 g/dL with an absolute increase of ≥0.5 g/dL, progression of involved serum FLC by ≥50% or the ratio of involved to uninvolved FLC ratio ≥100 when absolute FLC ratio is at least 100 mg/L, and urine protein electrophoresis showing urine M protein ≥500 mg in 24 hours. CBC, complete blood count; FLC, free light chain; IgG, immunoglobulin G; MGUS, monoclonal gammopathy of undetermined significance.

During these follow-up evaluations, laboratory studies focusing on the complete blood count, creatinine, and calcium should be commenced along with quantification of the serum M protein. Routine imaging studies and bone marrow assessment should not be done without clinical or laboratory “red flags,” which could signify disease progression. Figure 2 represents a flow diagram to summarize the workflow involved in monitoring patients with MGUS.

## Conclusion and future directions

MGUS is a precursor state for LPMs, including MM, AL, and WM. When patients with MGUS are evaluated, accurate assessment should be done to risk-stratify patients, as this will guide future monitoring. It is important to keep in mind that the majority of patients with MGUS will never progress to an aggressive malignancy during their lifespan, and having a diagnosis of a precancerous state could be a psychological burden for these patients. With the advent of genetic sequencing techniques such as WES, the field is rapidly evolving and new molecular and genetic signatures that will enable better and early risk stratification of MGUS are looming. With future studies, we hope to be able to better understand the pathobiology of MGUS that leads to disease progression and to be able to better risk-stratify patients and streamline the evaluation, thus improving the physical and psychological well-being of patients.
